# Schistosoma Infection Burden and Risk Factors among School-Aged Children in a Rural Area of the Democratic Republic of the Congo

**DOI:** 10.3390/tropicalmed8090455

**Published:** 2023-09-21

**Authors:** Sylvie Linsuke, Gillon Ilombe, Michel Disonama, Jean Deny Nzita, Placide Mbala, Pascal Lutumba, Jean-Pierre Van Geertruyden

**Affiliations:** 1 Department of Epidemiology, National Institute of Biomedical Research (INRB), Kinshasa 01015, Democratic Republic of the Congo; placide.mbala@inrb.cd; 2 Global Health Institute, Faculty of Medicine, University of Antwerp, 2000 Antwerp, Belgium; gillonilombe@yahoo.fr (G.I.); jean-pierre.vangeertruyden@uantwerpen.be (J.-P.V.G.); 3 Department of Parasitology, National Institute of Biomedical Research (INRB), Kinshasa 01015, Democratic Republic of the Congo; 4 Health Zone of Kwilu-Ngongo, Kongo-Central Province, Kwilu-Ngongo 20, Democratic Republic of the Congo; drmidiso3@gmail.com (M.D.); jdnzita@gmail.com (J.D.N.); 5 Department of Virology, University of Kinshasa, Kinshasa 01015, Democratic Republic of the Congo; 6 Department of Tropical Medicine, University of Kinshasa, Kinshasa 01015, Democratic Republic of the Congo; pascal_lutumba@yahoo.fr

**Keywords:** *Schistosoma* infection, prevalence, burden, risk factors, rural area, the Democratic Republic of the Congo

## Abstract

Despite continuous efforts to control schistosomiasis (SCH) in the Democratic Republic of the Congo (DRC), it still poses a significant challenge. In order to enhance control measures, additional research is necessary. This study documents the burden of SCH infection and its predictors in a rural area of the DRC. We conducted a household cross-sectional study from June to August 2021 among 480 school-aged children (SAC) aged 5–15 years living in a rural area of Kisangi, in the southwest DRC. We collected and examined stool, urine, and blood samples of each child. Additionally, we obtained data on anthropometry, socio-demographics, household information, and individual water contact behaviors. The overall prevalence of SCH infection was 55.8% (95% CI: 51.4–60.3), with prevalences of 41% (95% CI: 36.6–45.5), 36.3% (95% CI: 31.9–40.6), and 38.4% (95% CI: 32.6–44.3) for *S. haematobium* and *S. mansoni* infections and both infections, respectively. Among those with SCH infection, most had a light (67.5%) or heavy (51.7%) infection intensity. The geometric mean egg count was 16.6 EP 10 mL (95% CI: 12.9–21.3) for *S. haematobium* and 390.2 EPG (95% CI: 300.2–507.3) for *S. mansoni*. However, age (10 years and above (aOR: 2.1; 95% CI: 1.5–3.1; *p* < 0.001)) was an independent risk factor for SCH infection. The overall prevalence of malaria infection was 16.9% (95% CI: 13.5–20.2), that of stunting was 28.7% (95% CI: 24.7–32.8), that of underweight was 17.1% (95% CI: 12.8–21.4), and that of thinness was 7.1% (95% CI: 4.8–9.4). Anemia was prevalent at 49.4% (95% CI: 44.9–5), and the median Hb level of all participants was 11.6 g/dL (IQR: 10.5–12.6 g/dL). Anemia was strongly associated with SCH infection (aOR: 3.4; 95% CI: 2.3–5.1; *p* < 0.001) yet there was no association with the risk for malaria infection (aOR: 1.0; 95% CI: 0.6–1.8; *p* = 0.563). In addition, the risk of anemia increased with heavy infection intensities (*p* < 0.026 and *p* < 0.013 for *S. haematobium* and *S. mansoni*, respectively). However, stunting had a protective factor for anemia (aOR: 0.3; 95% CI: 0.2–0.4; *p* < 0.001). To conclude, SCH infection was widespread among the SAC and strongly linked to anemia. These results provide evidence of the hyperendemicity of infection in the study area, which requires preventative measures such as chemotherapy to reduce the schistosomiasis-associated morbidity, and micronutrient supplements to avoid anemia.

## 1. Introduction

Globally, approximately 779 million people worldwide are at risk of schistosomiasis infection, and 236.6 million required preventive chemotherapy in 2019 [1,2]. Sub-Saharan Africa (SSA) remains a public health problem, accounting for more than 90% of all infections globally [1]. Several factors, including poverty, the absence of or less access to improved water, sanitation, and hygiene (WASH) education, and environmental conditions, are reported to increase the risk of infection in endemic areas [3,4,5,6].

Humans acquire the infection when they come into contact with infected water with the larval forms of the parasite, often through fishing, agriculture, bathing, or performing certain daily domestic activities [7,8]. Adult worms live in the blood vessels where the females release eggs after coupling with male worms. Some eggs are evacuated in the feces or urine to continue the parasite’s life cycle; others become trapped in body tissues, stimulating immune reactions that can progressively damage organs [8,9]. In schoolchildren aged 5–15, chronic schistosomiasis can cause serious health problems, including malnutrition, anemia due to chronic inflammation and iron deficiency, impaired cognition, organ pathology, and others [10,11,12]. Additionally, schistosomiasis and malaria are very common in SSA and frequently result in common morbidities. Like *Schistosoma* infection, malaria infection has been associated with malnutrition, anemia, and organ pathology [13,14,15]. In endemic countries, the co-endemicity of both diseases has a high potential risk of increased morbidities in schoolchildren, particularly in terms of anemia morbidity, which reduces cognitive potential, retards growth, and predisposes schoolchildren to other diseases [16,17,18].

To control and eliminate schistosomiasis as a public health problem, the World Health Organization (WHO) promotes preventive chemotherapy through mass drug administration with praziquantel for all school-aged children (SAC), the treatment of at-risk adults in highly endemic areas, and additional interventions, including access to the WASH system and snail control [19]. Preventive chemotherapy remains the core public health strategy, which aims to reduce the prevalence and intensity of infection, prevent severe morbidity, and/or reduce other schistosomiasis-related morbidities [19]. However, before initiating any intervention, the WHO recommends that information on the endemicity of schistosomiasis and its potential impacts on health be established from the target group [20]. This information could be essential to categorize at-risk populations and determine the frequency of the intervention. In the Democratic Republic of the Congo (DRC), schistosomiasis remains a major health problem [16,21,22], particularly in rural communities, where the majority of the population is poorer and environmental conditions increase the risk [23]. Based on various data, the DRC contributed to 15 million cases of all reported schistosomiases cases worldwide and was ranked with Ghana as the third country in SSA with the highest burden of SCH [16,24]. Data from several epidemiological studies conducted in many parts of the country, especially among the SAC population, confirmed these estimates and place the DRC among the countries requiring preventive chemotherapy [25,26,27,28]. The National Schistosomiasis Program adopted preventive chemotherapy as the primary intervention strategy to reduce the burden of infection. Since 2015, initiatives have been launched against schistosomiasis. As recommended by the WHO guidelines [29], the baseline prevalence data were obtained from a nationwide survey [30], and preventive chemotherapy was implemented in some endemic areas of the country. Meanwhile, observation of routine laboratory activities in many other rural areas revealed a substantial number of important suspects in *Schistosoma* infection. The problem appears to be worse, which would argue for integrating preventive chemotherapy intervention into the primary health care systems (PHC systems) in these areas. Unfortunately, without more evidence to support this observation, it is impossible to integrate preventive chemotherapy in these areas. We emphasize the need for more scientific evidence on the burden of infection for decision making to optimize control strategies in the decades to come. This study aimed primally to document the burden of *Schistosoma* infection and its predictors among SAC living in the rural area of Kisangi in the southwest of the DRC. Meanwhile, we also investigated the malaria infection status and explored the anemia and nutritional statuses of schoolchildren, considering the overlap between schistosomiasis and malaria infections in the study area.

## 2. Materials and Methods

### 2.1. Study Setting

**Figure 1:**
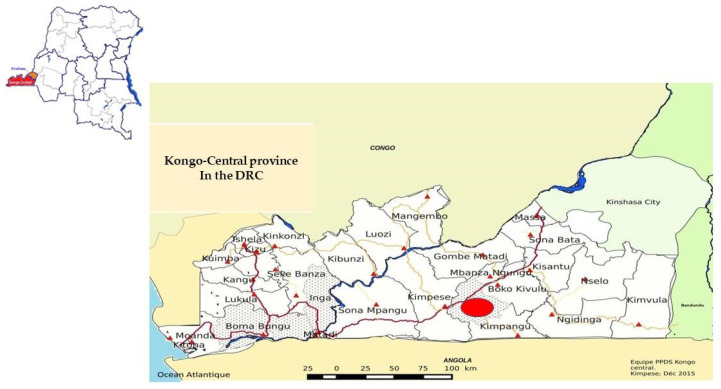
Kongo Central Health Map in the DRC indicating the study site in red.

The study was conducted from June to August 2021 in Kisangi village located in the Kwilu-Ngongo Health Zone (province of Kongo Central) in the southwest of the DRC between 5°36′7 degrees latitude south and 14°35′6 degrees longitude east (Figure 1). This study area has a tropical climate with a rainy season from September 15 to May 15 and a dry season from May 15 to September 15 of each year, interrupted by a short dry season in February. The period of our study corresponded to the dry season, during which transmission is highest. According to information obtained from the health zone, the population of the area was estimated at approximately 5.536 inhabitants in 2021, out of which 1445 were SAC, and there are 4 primary schools in the area (report archive of the health zone). Agriculture represents the main activity of the population, although a few inhabitants fish. In addition, an immense plantation of sugar cane surrounds the health area. Households have no access to a piped water supply in the village. Inhabitants of the area have access to clean water through improved supply sources; otherwise, they have to obtain it from the nearest river and from water drilling, which is located approximately 1.5 km outside the village. Moreover, agriculture is practiced throughout all seasons and the population is permanently exposed to the infection through domestic (washing clothes and dishes and performing other activities in the river) and/or irrigation activities.

Based on the health zone report, there is an important burden of *Schistosoma* infection in the area, but no preventive activities for control had previously been made in the area before our study, and only a few enrolled SAC received albendazole at primary school in 2019 (report archive of the health zone). In addition, our study area was endemic for malaria infection. The decision to choose this area was based on the substantial number of cases reported, but the study area is not yet included in the preventive chemotherapy intervention.

### 2.2. Study Design and Procedure

This was a household-based cross-sectional study that involved the SAC population aged 5 to 14 years who lived in the selected village during the survey. Before the survey and recruitment, the research team visited the households and informed the heads of the households of the objects and the details of the study. The local health workers also mobilized the village to ensure a high participation rate. All eligible children who agreed to participate in the study and those whose parents or legal guardians gave written informed consent were recruited via door-to-door census. From each child, one stool sample and one urine sample were collected and tested for the presence and intensity of helminth infections. Simultaneously, finger-prick blood samples were collected from each enrolled child to assess malaria infection and hemoglobin (Hb) levels. In addition, demographic (sex and age), nutritional parameter (weight and height), household information (presence of taps and toilet), and individual water contact behavior data were also collected using a structured questionnaire to assess factors associated with *Schistosoma* infection.

### 2.3. Laboratory Analysis

Diagnosis of *Schistosoma* infection: Stool and urine samples were collected in the early morning and shipped in an ice box to the laboratory of IME Kimpese for preparation and analysis, according to the standard operating procedures of the study. For each stool sample, duplicate Kato–Katz thick smears (2 × 25 mg) [31] were prepared and examined by two trained technicians 24 h later to detect *S. mansoni* eggs and other intestinal helminths, such as *A. lombricoides* and *T. trichiura* eggs; however, the results reported in the present study were only focused on *Schistosoma* infection. Urine samples were analyzed for the presence of *S. haematobium* eggs using a standard filtration technique, as described previously [32]. The Schistosoma species infection intensity was determined for positive samples. According to WHO criteria [30] for infection intensity, *S. mansoni* was quantified as the number of eggs per gram (EPG) of feces calculated from a template of 25 mg of feces and was then graded into low (1–99 EPG), moderate (100–399 EPG), and heavy infection (≥400 EPG), while *S. haematobium* was quantified as the number of eggs per 10 mL of urine filtered (eggs/10 mL) and then graded into low (1–49 eggs) and heavy (≥50 eggs). Likewise, *Schistosoma* infection was interpreted as positive with at least one egg of either *S. mansoni* found in the stool samples or *S. haematobium* in the urine samples, or as negative in the absence of eggs in the two samples tested. Monoinfection was defined as an infection of only one species, while mixed infection was defined as an infection with two *Schistosoma* species.

Diagnosis of malaria infection: Thick and thin blood films were prepared for each collected blood sample on the same slide. According to a previously described procedure [33], the slides were fixed and stained for 10 min with 10% (1.9 mL) Giemsa stain. Light microscopy at a magnification of 1000× was used for examination of the blood slides, and then parasite density was calculated per microliter of blood using the following formula: (Number of trophozoites × 8000)/Number of leucocytes. Parasite counts were used to classify the density of *Plasmodium falciparum* (*P. falciparum*) infection into light (1–499 parasites/µL), moderate (500–1.999 parasites/µL), and heavy (2.000–9.999 parasites/µL).

Determination of Hb levels: Hb levels were measured in g/dL using a HemoCue Analyzer Hb 301 portable (HemoCue Hb 301, EKF Diagnostics-GmbH, Barleben, Germany), according to the manufacturer’s instructions. Anemia was defined according to the WHO cut-offs adjusted for age and gender as Hb < 11.5 g/dL (children aged 5–11 years), Hb < 12.0 g/dL (children aged 12–14 years), and Hb < 12.0 g/dL (for girls aged 15 years old). Likewise, the gravity of anemia was classified as mild (10–11.4 g/dL), moderate (7–9.9 g/dL), and severe (<7 g/dL) [34].

Anthropometric measurements: Anthropometric measurements were performed by a trained nurse assisted by a community health worker to assess the nutritional status of the schoolchildren. The weight was determined in kilograms in decimal increments with a digitally calibrated portable balance (Seca 888, Seca GmbH & co.kg., Hamburg, Germany), and the height in centimeters, up to the decimal, was determined with a portable stadiometer (Seca 225, Seca GmbH, Germany) with a stand anthropometric position arrow bounding the height of the subject. These values were used to calculate the height-for-age Z-score (HAZ), weight-for-age Z-score (WAZ), and body mass index-for-age Z-score (BAZ) using WHO AnthroPlus software 1.0.4 [35]. Weight, height, and BMI values were related to age. According to the WHO growth reference for SAC and adolescents [36], children were categorized as stunted, underweight, or thin. 

Quality control: All slides of Kato–Katz thick smears and thick and thin blood films were independently read by two trained technicians. In case of disagreement between the readers, slides were reviewed by a senior technician who decided and confirmed the diagnosis. Furthermore, a senior technician re-examined ten percent of the randomly selected slides as quality control.

### 2.4. Statistical Analysis

Data from this study were double-entered into Epi-Info 3.1.5. software (CDC, Atlanta, GA, USA) and analyses were performed using the Stata program, version 11.1, for Windows (Stata Corp., College Station, TX, USA). Descriptive statistics were used to summarize the data. Continuous data were described using the mean ± standard deviation (SD) or median and interquartile range (IQR), depending on the distribution. Frequencies and proportions with corresponding 95% confidence intervals (CIs) were used to describe categorical variables. Bivariate logistic regression analysis was applied to identify the factors associated with *Schistosoma* infection. The odds ratios (ORs) and their corresponding 95% CIs were reported for each variable, and *p*-values less than 0.05 were considered statistically significant. Multivariate logistic regression models were constructed to identify factors associated with *Schistosoma* infection and anemia among schoolchildren. The backward regression technique was used to build the model.

### 2.5. Ethics Approval and Consent to Participate

The present study was conducted in full agreement with the principles of the Declaration of Helsinki. Ethical approval was obtained from the National Ethics Committee of Health, Kinshasa (DRC), approval code n’266/CNES/BN/PMMF/2021. We also obtained administrative authorization from the DRC Ministry of Health, the provincial Ministry of Health, and the local authorities of the village. Written informed consent from a parent or legal guardian and the assent of the SAC were obtained before recruitment. At the end of the parasitological survey, all SAC were treated with a single dose of 40 mg/kg praziquantel (600 mg) and a single dose of albendazole (400 mg) for the treatment and prevention of schistosomiasis and soil-transmitted helminths (STHs), respectively [30]. Children who tested positive for malaria infection were advised to visit the nearest health center when they developed fever for the treatment of malaria, according to national policy.

## 3. Results

### 3.1. General Characteristics of Study Participants

The general characteristics of all the participants are presented in Table 1. The study enrolled 480 schoolchildren, of whom 50.2% (241/480) were males. The median age of all participants was 9 years (interquartile range (IQR) = 7–11). Most of the schoolchildren (251 (52.3%)) were aged between 5 and 9 years old. None of the study participants reported having access to water in their households, and 24.4% (95% CI: 20.5–28.2) reported having a toilet. They use the nearest river for domestic activities. Approximately ninety percent (89.2%; 95% CI: 86.4–91.9) reported having regular contact with water.

### 3.2. Schistosoma Infection

The overall prevalence of *Schistosoma* infection among the enrolled schoolchildren in the study was 55.8% (95% CI: 51.4–60.3). *S. haematobium* infection was found in 197 (41%; 95% CI: 36.6–45.5) schoolchildren, *S. mansoni* in 174 (36.3%; 95% CI: 31.9–40.6), and 103 (38.4%; 95% CI: 32.6–44.3) out of 268 infected schoolchildren had both infections (Table 1).

The *Schistosoma* infection intensities are summarized in Table 2. Out of those infected with *S. haematobium*, 67.5% had light infection intensities while 32.5% had heavy infection intensities. The geometric mean egg count was 16.6 EP 10 mL (95% CI: 12.9–21.3). The infection intensity for *S. mansoni* infection was recorded as light (21.3%), moderate (27.0%), and heavy (51.7%). The geometric mean egg count for those infected was 390.2 EPG (95% CI: 300.2–507.3).

In the multivariate logistic regression (Table 3), we found that *Schistosoma* infection was associated with age (aged 10 years and above (aOR: 2.1; 95% CI: 1.5–3.1; *p* < 0.001)). Other habitual evaluated risk factors for *Schistosoma* infection, such as sex, swimming in the river, washing clothes in the river, washing dishes in the river, and the presence of taps and toilets in the households, were not significantly associated with *Schistosoma* infection.

### 3.3. Malaria Infection

Of the 480 blood samples screened for malaria infection, 81 were positive, representing an overall prevalence of malaria infection of 16.9% (95% CI: 13.5–20.2) (Table 1). Of the different species of *Plasmodium* identified among the infected schoolchildren, 14.6% (95% CI: 11.4–17.7) were *P. falciparum* and 2.1% (95% CI: 0.8–3.4) were *P. malariae*. Only one case (0.2%; 95% CI: 0.0–0.01) of mixed *P. falciparum–P. malariae* infection was observed among the malaria-infected schoolchildren (Table 1). In addition, the malaria infection density was found to be light at 5.2%, moderate at 5.6%, and heavy at 3.9, with the median parasite density estimated to be 927.4/µL (95% CI: 667.4–1288.7) (Table 2).

### 3.4. Nutritional Status

As shown in Table 1, approximately 138 (28.7%; 95% CI: 24.7–32.8) of the schoolchildren were stunted, with a mean HAZ of −1.7 ± 1.6; 51 (17.1%; 95% CI: 12.8–21.4) were underweight, with a mean WAZ of −1.5 ± 1.2; and 34 (7.1%; 95% CI: 4.8–9.4) were thin, with a mean BAZ of −0.9 ± 1.3. Underweight was calculated only for children < 10 years: n = 298.

### 3.5. Anemia

The median Hb level of all the schoolchildren was 11.6 g/dL (IQR: 10.5–12.6 g/dL). The prevalence of anemia in the study was 49.4% (95% CI: 44.9–53.9). Of the anemic schoolchildren, 64 (27%; 95% CI: 21.3–32.7), 166 (70%; 95% CI: 64.2–75.9), and 7 (2.9%; 95% CI: 0.08–0.5) had mild, moderate, and severe anemia, respectively (Table 1). Among the anemic schoolchildren, 164 (61.2%) also had *Schistosoma* infection, and 36 (44.4%) had malaria infection (Table 4). Moreover, in the multivariate regression analysis (Table 4), anemia was associated with *Schistosoma* infection (aOR: 3.4; 95% CI: 2.3–5.1; *p* < 0.001), and there was no association between anemia and malaria infection (aOR: 1.0; 95% CI: 0.6–1.8; *p* = 0.563). In addition, the risk of developing anemia was lower among schoolchildren aged 10 years and above (aOR: 0.6; 95% CI: 0.4–0.9; *p* = 0.012) and those with stunting (aOR: 0.3; 95% CI: 0.2–0.4; *p* < 0.001).

### 3.6. Schistosoma Species and Malaria Infection Intensities Correlate to Hb Levels

**Figure 2:**
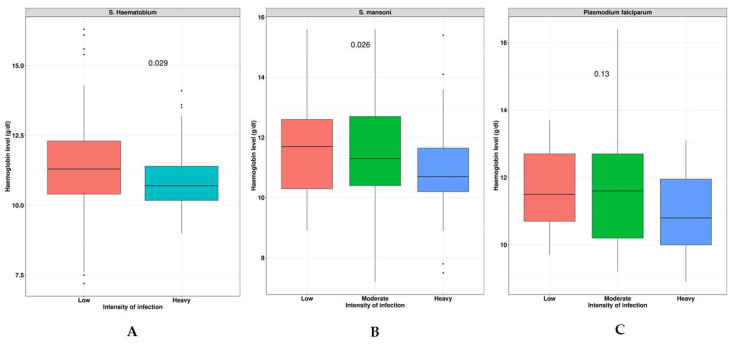
Effects of *S. haematobiun*, *S. mansoni*, and malaria infection intensities on Hb levels. Panel (**A**): indicates the severity of *S. haematobium* infection correlates with anemia; Panel (**B**): the severity of *S. mansoni* infection correlates with Hb level; and Panel (**C**): the severity of malaria infection correlates with Hb level.

The study assessed the relationships between the *S. haematobium*, *S. mansoni*, and malaria infection intensities on the Hb levels (as shown in Figure 2). The findings revealed that individuals with a heavy *S. haematobium* infection (*p* < 0.029) were more likely to have anemia compared to those with a low infection intensity (as seen in Figure 2A). Additionally, those with a heavy *S. mansoni* infection (*p* < 0.026) were more likely to develop anemia than those with a low or moderate infection intensity (as shown in Figure 2B). However, no significant effect on the Hb levels was observed for the malaria infection intensity (*p* < 0.013) (as shown in Figure 2C).

## 4. Discussion

We found, in the SAC, an overall prevalence of *Schistosoma* infection at 55.8%, malaria infection at 16.9%, stunting at 28.7%, underweight at 17.1%, thinness at 7.1%, and anemia at 49.4%, with a median Hb level of 11.6 g/dL (IQR: 10.5–12.6 g/dL). The study also revealed an association between age and *Schistosoma* infection (aged 10 years and above (aOR: 2.1; 95% CI: 1.5–3.1; *p* < 0.001)), but there were no associations found between *Schistosoma* infection and other habitual-related risk factors, such as gender, water contact, and sanitation. Furthermore, in this study, *Schistosoma* infection contributed highly to the anemia (aOR: 3.4; 95% CI: 2.3–5.1; *p* < 0.001) in the SAC. However, the risk of having anemia was highest for individuals suffering from a heavy infection intensity (*p* < 0.026 and *p* < 0.013 for *S. haematobium* and *S. mansoni*, respectively). No association with the risk of anemia was found for malaria infection.

The prevalence of *Schistosoma* infection observed in this study was high, similar to those reported in previous studies conducted in different parts of the DRC [28,37,38], but it was relatively lower than those previously reported in Kasansa (82.7%) [25] and Ituri (ranging from 59.2 to 76.6%) [39]. A similar prevalence was also reported elsewhere [40,41]. However, this prevalence was higher than those reported in other parts of the DRC [27,42,43,44] and other SSA countries [45,46]. The lack of public health interventions for this infection contributed highly to the high rate of *Schistosoma* infection observed in our study. This indicates that *Schistosoma* infection is still a considerable public health problem concerning the SAC population living in the area. Hence, it is crucial to implement preventive chemotherapy for this community, especially for all SAC.

This study found that age was an independent risk factor for *Schistosoma* infection. Participants aged 10–14 years were at a higher risk of infection than those aged less than 10 years, similar to the studies from the DRC [38,39] and elsewhere [46,47,48,49]. The main target risk group for *Schistosoma* infection consists of SAC of this age group due to their vulnerability to infection because of their behavior. Indeed, SAC aged 10–14 often spend most of their time at the river for recreational activities or to collect household water supplies. This situation increases their likelihood of contracting the infection [4,50].

In contrast, other risk factors studied in our study, such as gender, swimming, washing clothes, washing dishes in the river, and the presence of taps and toilets in the households, did not show significant associations with *Schistosoma* infection. This finding contradicts previous studies conducted elsewhere [27,39,48,51]. However, as for our study, a recent study from Tanzania [52] also did not show an association between *Schistosoma* infection and water contact, although most participants had regular water contact. Nevertheless, the occurrence of infection in endemic areas depends on different components, such as climate, environmental factors, the ecological conditions across the area, and sociocultural factors that determine personal behavior [53,54]. The insignificant association between *Schistosoma* infection and gender, water contact, and the sanitation conditions observed in our study could probably be due to the different levels of exposition between SAC, even in the same village. There is a need to document this level of environmental exposition. However, for gender, girls and boys could be exposed at the same level, although the data from this study did not demonstrate this association.

In this study, we estimated the prevalence of malaria infection at 16.9%. A similar result was found in the Biyela health zone, DRC [43]. However, this contrasted with the result from Kasansa [26], in which the author reported a high prevalence of 65.1% in SAC. These conflicting results on the prevalence of malaria infection observed in these different studies could probably be explained by the seasonal variability in the transmission of malaria infection.

Our study also estimated the nutritional statuses of the SAC, which were stunted at 28.7%, underweight at 17.1%, and thinness at 7.1%. This was in disagreement with the study from Biyela [43], in which the author reported lower prevalences of 0.16%, 0.97%, and 0.65%, respectively, for stunting, underweight, and thinness. Meanwhile, a previous study from Ghana [55] also reported similar proportions of nutritional statuses to those reported in our study. Likewise, children presenting stunting were protected from the risk of developing anemia (aOR: 0.3; 95% CI: 0.2–0.4; *p* < 0.001). This result is paradoxical and may be a confounding factor, as malnutrition is an important aspect of the risk of anemia. Unfortunately, our study did not analyze the effect of infection on malnutrition. Further studies including several factors that explain this situation are needed.

In this study, the prevalence of anemia was 49.4%, which was found to be lower than those reported in Kasansa [26] and Lemfu [42], but it was closer to those of Biyela [43]. In addition, a close association was observed in this study between anemia and *Schistosoma* infection. Compared to other studies in different parts of the country, this result is consistent with those from Biyela [43]. However, it differed from the finding from Kasansa [40], although an important prevalence of anemia was observed among SAC living in this area. Our result also contrasted those previously reported in Tanzania, where authors reported no association between schistosomiasis and anemia [40,56,57]. For those authors, different factors, such as the dependance on food availability and the endemicity for other parasitic infections such as malaria and hookworm in the regions, contributed highly to anemia among schoolchildren. Indeed, the relationship between schistosomiasis and anemia was documented previously [11], and this finding further highlights the importance of schistosomiasis as a contributor to anemia in SAC. In contrast, no association was found in this study between anemia and malaria infection. This finding is not in keeping with the finding from Biyela [43], which reported the simultaneous association of anemia with malaria and schistosomiasis, suggesting that both parasites contributed highly to anemia among SAC living in endemic areas.

However, heavy *Schistosoma* infection intensities (*S. haematobium*; *p* < 0.026 and *S. mansoni*; *p* < 0.013) increased the risk of anemia in this study. This concurs with studies from Uganda [58] and Ethiopia [59], while it contrasts with the study from Mali [60]. This result suggests that anemia may serve as an important indicator of morbidity resulting from severe infection.

Although this study has provided valuable data for the control program to integrate preventive chemotherapy in this area, it has some limitations. First, we collected only one stool sample and one urine sample from each study participant, while some studies have recommended a more substantial sample collection of three consecutive days to increase the sensitivity of the different techniques. It is possible that the observed prevalence of infection in this study could be underestimated. Nevertheless, this did not impact our results, as the observed prevalence indicates that schistosomiasis remains a major public health problem in this area. Also, infection with hookworm was not tested in this study, as all slides were examined 24 h after their preparation. Thus, we did not test for the contribution of hookworm to anemia among SAC living in this area. Yet, hookworm could play an important role in the development of anemia, as for *Schistosoma* infection and/or malaria. Nevertheless, given the environmental conditions of this area, we think hookworm is not very prevalent in the area to the point that it leads to anemia in children. Further studies that estimate the prevalence and influence of hookworm on Hb levels are needed. Another limitation of our study was that a risk factor analysis for malaria and the nutritional status of the schoolchildren was not performed in our study due to the lack of adequate variables. Because this was not the primary focus of our study, further studies are needed.

## 5. Conclusions

This study reports a high prevalence of *Schistosoma* infection among SAC living in the rural area of Kisangi in the southwest of the DRC, in which the prevalence was highly associated with anemia resulting from the high burden of infection. Given this high prevalence, this study site is classified as a hyperendemic area for *Schistosoma* infection according to WHO guidelines [29]. Hence, we highlight the need for integrated control approach strategies to reduce the burden of this disease in this area. These strategies include preventive chemotherapy with praziquantel in this community, especially among SAC, to reduce the infection and related morbidity. Micronutrient supplementation of the SAC to avoid anemia is also essential.

## Figures and Tables

**Table 1:** General characteristics of study participants.

Variables	Frequency	% (95% CI)
Overall	480	
Gender		
Female	239	49.8 (45.3–54.3)
Male	241	50.2 (45.7–54.7)
Median age (IQR)	9 (7–11)	
Age groups (years)		
5–9	251	52.3 (47.8–56.8)
10–14	229	47.7 (43.2–52.2)
Household sanitation		
Toilet in the household	117	24.4 (20.5–28.2)
Taps in the household	480	100
Water contact	428	89.2 (86.4–91.9)
Swimming in the river	382	79.6 (75.9–83.2)
Washing dishes in the river	281	58.5 (54.1–62.9)
Washing clothes in the river	329	68.5 (64.4–72.7)
Parasitic infection		
*Schistosoma* infection	268	55.8 (51.4–60.3)
*S. haematobium*	197	41.0 (36.6–45.5)
*S. mansoni*	174	36.3 (31.9–40.6)
Mixed *S. haematobium–S. mansoni*	103	38.4 (32.6–44.3)
Malaria infection	81	16.9 (13.5–20.2)
*P. falciparum*	70	14.6 (11.4–17.7)
*P. malariae*	10	2.1 (0.8–3.4)
Mixed *P. falciparum–P. malariae*	1	0.2 (0.0–0.01)
Coinfection *Schistosoma*–malaria	31	6.5 (4.3–8.7)
Median Hb level, g/dL (IQR)	11.6 (10.5–12.6)	
Anemia	237	49.4 (44.9–53.9)
Mild anemia (Hb 10–11.4 g/dL)		27.0 (21.3–32.7)
Moderate anemia (Hb 7–9.9 g/dL)	166	70.0 (64.2–75.9)
Severe anemia (Hb < 7 g/dL)	7	2.9 (0.08–0.5)
Nutritional status		
Stunting	138	28.7 (24.7–32.8)
HAZ, mean ± SD	−1.7 ± 1.6	
Underweight	51	17.1 (12.8–21.4)
WAZ, mean ± SD	−1.5 ± 1.2	
Thinness	34	7.1 (4.8–9.4)
BAZ, mean ± SD	−0.9 ± 1.3	

**Table 2:** Parasite infection intensities.

Variables	*S. haematobium*	*S. mansoni*	*P. falciparum*
No. of non-infected (%)	283 (58.9)	306 (63.8)	409 (85.2)
No. of light infection (%)	133 (67.5)	37 (21.3)	25 (5.2)
No. of moderate infection (%)	NA	47 (27.0)	27 (5.6)
No. of heavy infection (%)	64 (32.5)	90 (51.7)	19 (3.9)
Geometric mean egg count (95% CI)	16.6 (12.9–21.3)	390.2 (300.2–507.3)	927.4 (667.4–1288.7)

**Table 3:** Risk factors for *Schistosoma* infection among schoolchildren from the Kisangi area.

Variables	N	n (%)	cOR (95% CI)	*p*-Value	aOR (95% CI)	*p*-Value
Gender						
Female	239	136 (56.9)	Ref.			
Male	241	132 (54.8)	0.9 (0.6–1.3)	0.638	0.9 (0.6–1.3)	0.638
Age groups (years)						
5–9	251	118 (47.0)	Ref.			
10–14	229	150 (65.5)	2.1 (1.5–3.1)	<0.001	2.2 (1.5–3.1)	<0.001 *
Swimming in the river						
No	98	53 (54.1)	Ref.			
Yes	382	215 (56.3)	1.1 (0.7–1.7)	0.696	1.1 (0.7–1.8)	0.594
Washing clothes in the river						
No	151	88 (58.3)	Ref.			
Yes	329	180 (54.7)	0.9 (0.6–1.3)	0.465	0.7 (0.4–1.3)	0.253
Washing dishes in the river						
No	199	111 (55.8)	Ref.			
Yes	281	157 (55.9)	1.0 (0.7–1.4)	0.984	1.3 (0.7–2.1)	0.399
Taps in the household						
No	480	268 (55.8)	Ref.			
Yes	0	0 (0)	(omitted)	–	–	–
Toilet in the household						
No	363	203 (55.9)	Ref.			
Yes	117	65 (55.6)	1.0 (0.6–1.5)	0.945	1.0 (0.7–1.6)	0.925

* Significant at *p* ≤ 0.05, cOR = crude odds ratio, aOR = adjusted odds ratio.

**Table 4:** Predictors for anemia among schoolchildren from the Kisangi health area.

Variables	N	n (%)	cOR (95% CI)	*p*-Value	aOR (95% CI)	*p*-Value
Gender						
Female	239	121 (50.6)	Ref.			
Male	241	116 (48.1)	0.9 (0.6–1.3)	0.585	0.9 (0.6–1.4)	0.785
Age groups (years)						
5–9	251	133 (52.9)	Ref.			
10–14	229	104 (45.4)	0.7 (0.5–1.1)	0.098	0.6 (0.4–0.9)	0.012 *
*Schistosoma* infection						
No	212	73 (34.4)	Ref.			
Yes	268	164 (61.2)	3.0 (2.1–4.4)	<0.001	3.0 (2.0–4.6)	<0.001 *
Malaria infection						
No	399	201 (50.4)	Ref.			
Yes	81	36 (44.4)	0.8 (0.5–1.3)	0.331	1.0 (0.6–1.8)	0.563
Stunting						
No	342	201 (58.8)	Ref.			
Yes	136	36 (26.1)	0.2 (0.2–0.4)	<0.001	0.3 (0.2–0.4)	<0.001 *
Underweight						
No	247	137 (55.5)	Ref.			
Yes	51	22 (43.1)	0.6 (0.3–1.1)	0.110	–	-
Thinness						
No	446	222 (49.8)	Ref.			
Yes	34	15 (44.1)	0.8 (0.4–1.6)	0.525	–	-

* Significant at *p* ≤ 0.05. cOR: crude odds ratio; aOR: adjusted odds ratio.

## Data Availability

The authors confirm that all data supporting the findings are fully accessible and available without any restrictions. All relevant data supporting the findings of this study are included in the paper and its supporting information files.
